# Health System Enablers and Barriers to Continuity of Care for First Nations Peoples Living with Chronic Disease

**DOI:** 10.5334/ijic.7643

**Published:** 2023-12-11

**Authors:** Maria Alejandra Pinero de Plaza, Lemlem Gebremichael, Shannon Brown, Chiung-Jung Wu, Robyn A. Clark, Katharine McBride, Sonia Hines, Odette Pearson, Kim Morey

**Affiliations:** 1 Caring Futures Institute, College of Nursing and Health Sciences, Flinders University, Adelaide, SA, 5000, AU; 2 The Mparntwe Centre for Evidence in Health, Flinders University: A JBI Centre of Excellence. Alice Springs, NT, 0871, AU; 3 Library, Flinders University Adelaide, SA, 5000, AU; 4 School of Health, University of the Sunshine Coast, Petrie, QLD, 4502, AU; 5 Royal Brisbane & Women’s Hospital, QLD, 4029, AU; 6 South Australian Aboriginal Chronic Disease Consortium, Adelaide, SA, 5001, AU; 7 Wardliparingga Aboriginal Health Equity Theme, South Australian Health and Medical Research Institute, Adelaide, SA, 5001, AU; 8 Telethon Kids Institute, Adelaide, SA, 5000, AU; 9 The John Curtin School of Medical Research, The Australian National University, Acton, ACT 2601, AU; 10 Flinders University, Rural and Remote Health, Alice Springs, Northern Territory, 0871, AU; 11 Adelaide Medical School, The University of Adelaide, SA, 5000, AU

**Keywords:** Integration Of Care, Chronic Diseases, Aboriginal, Indigenous, First Nations People, Policymaking

## Abstract

**Introduction:**

Failings in providing continuity of care following an acute event for a chronic disease contribute to care inequities for First Nations Peoples in Australia, Canada, and Aotearoa (New Zealand).

**Methods:**

A rapid narrative review, including primary studies published in English from Medline, Embase, PsycINFO, and Cochrane Central, concerning chronic diseases (cancer, cardiovascular disease, chronic kidney disease, diabetes, and related complications), was conducted. Barriers and enablers to continuity of care for First Nations Peoples were explored considering an empirical lens from the World Health Organization framework on integrated person-centred health services.

**Results:**

Barriers included a need for more community initiatives, health and social care networks, and coaching and peer support. Enabling strategies included care adapted to patients’ cultural beliefs and behavioural, personal, and family influences; continued and trusting relationships among providers, patients, and caregivers; and provision of flexible, consistent, adaptable care along the continuum.

**Discussion:**

The support and co-creation of care solutions must be a dialogical participatory process adapted to each community.

**Conclusions:**

Health and social care should be harmonised with First Nations Peoples’ cultural beliefs and family influences. Sustainable strategies require a co-design commitment for well-funded flexible care plans considering coaching and peer support across the lifespan.

## Introduction

In Australia, Aboriginal and Torres Strait Islander Peoples experience inequitable healthcare services across the continuum of care, from primary prevention to tertiary care [1]. Chronic conditions are responsible for more than two-thirds (70%) of the gap in disease burden between Aboriginal and Torres Strait Islander Peoples and non-Indigenous Australians; [2] e.g., Aboriginal and Torres Strait Islander Peoples are admitted to hospitals for potentially preventable chronic diseases at a rate of 3.2 times higher than non-Indigenous Australians [3]. This gap can be understood and tackled via better continuity of care, which is defined by The World Health Organization (WHO) as the degree to which people experience a series of discrete healthcare events as coherent and interconnected over time and in a way that is consistent with their health needs and preferences [4].

**Figure 1:**
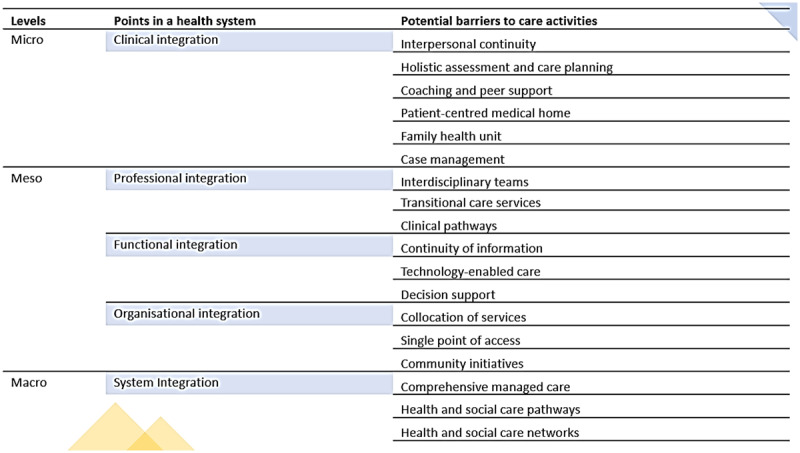
The points in a health system at which barriers to care continuity and its coordination can exert an influence – adapted from WHO’s Integrated People-Centred Health Services- [4].

Continuity of care is achieved when care coordination focuses on the conditions and the ongoing relationships needed to support harmonious interactions among multiple providers within interdisciplinary teams across care settings and sectors [4]. In such a continuum, the priority practices and actions enabling care integration at different levels are considered within the Rainbow model of integrated care [4, 5], which helps identify the points at which continuity of care and its coordination influence practice [4]. Such points are presented in Figure 1 as potential guidance for detecting barriers around continuity and integration of care considering the WHO’s Framework on Integrated People-Centred Health Services (IPCHS) and evidence-based classifications around its implementation [4, 5].

**Figure 2:**
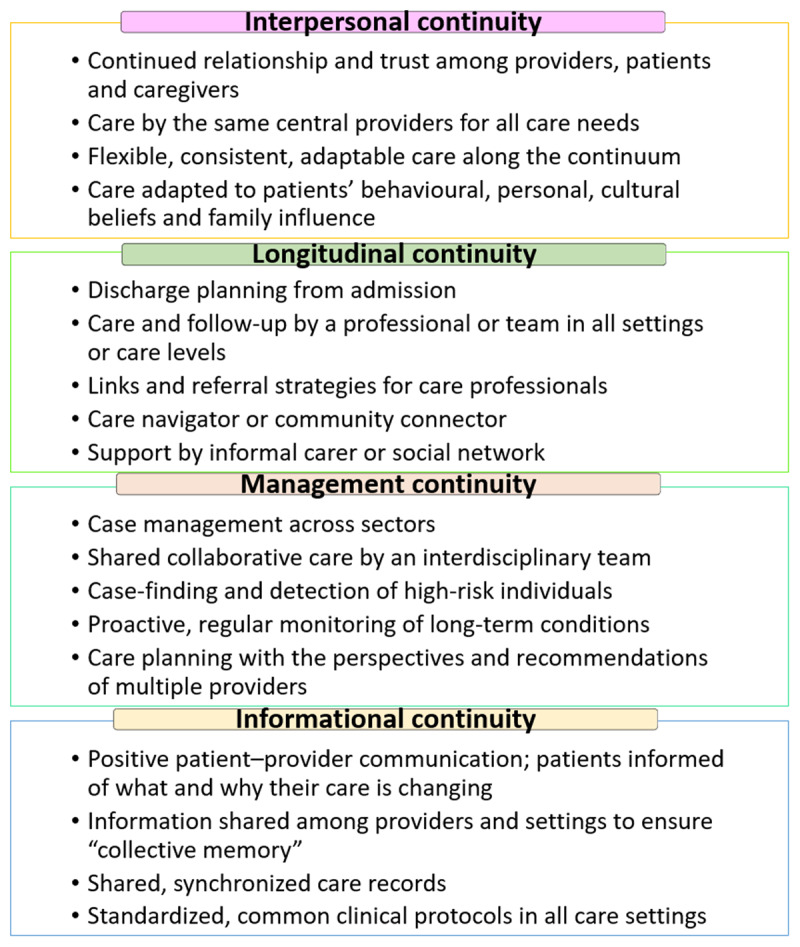
The range of approaches and interventions for achieving continuity of care (adapted from WHO’s Integrated People-Centred Health Services) [4].

High continuity of care translates into health benefits, including fewer emergency department visits, hospital admissions, and lower care costs [4]. Health professionals’ perspectives on communication and continuity of care for improving cancer care for Aboriginal and Torres Strait Islander Peoples in Queensland, Australia, recommended that communication, collaboration, and care coordination strategies be incorporated to generate health policy and funding across services and settings [6]. The WHO has also identified implementation approaches and interventions for improving continuity of care (Figure 2) [4], which can be used as a practical lens to comprehend current enablers to continuity of care and help bridge gaps for First Nations Peoples [4].

Achieving continuity of care is vital for addressing the existing shortcomings in healthcare delivery and addressing disparities faced by First Nations Peoples worldwide [7, 8]. These disparities are evident in First Nations populations in Australia, Canada, and New Zealand (Aotearoa hereafter), who share a history of colonisation and face common drivers of health inequities, including a high prevalence of chronic diseases despite having universal healthcare systems [7,8,9,10]. Across these three countries, First Nations Peoples’ understanding of health and wellbeing, whilst each being culturally specific, are holistic, interconnected, strength-based, and extend beyond the Western biomedical understandings of disease and illness by which their healthcare systems are designed on [11].

To comprehend and address the presented challenges, this review leverages real-world implementation strategies outlined in WHO’s IPCHS framework to synthesise empirical knowledge, providing insights into the barriers and facilitators of continuity of care from a health system perspective [4]. This review was initiated by the South Australian (SA) Aboriginal Chronic Disease Consortium [12], demonstrating their commitment to prioritising evidence-based approaches that promote comprehensive chronic disease prevention, management, and care [7, 8]. The Consortium is an Aboriginal-led partnership of health stakeholders that drives the delivery of collaborative, appropriate, well-coordinated and evidence-based strategies to reduce the burden of heart disease, cancer and diabetes [7, 8].

### Review question

What are the health system enablers and barriers to continuity of care for First Nations Peoples living with chronic conditions? [12]

### Methods

A rapid qualitative review was co-designed with leaders of the Wardliparingga Aboriginal Health Equity Theme at the South Australian Health and Medical Research Institute (SAHMRI) and the SA Aboriginal Chronic Disease Consortium following JBI and Cochrane reporting principles [7, 8, 12, 13]. During the review, consideration and revisions [7, 8, 12] were made to the a priori protocol registered in PROSPERO ID: CRD42022339990 (as reflected below) [12].

### Search Strategy

**Figure 3:**
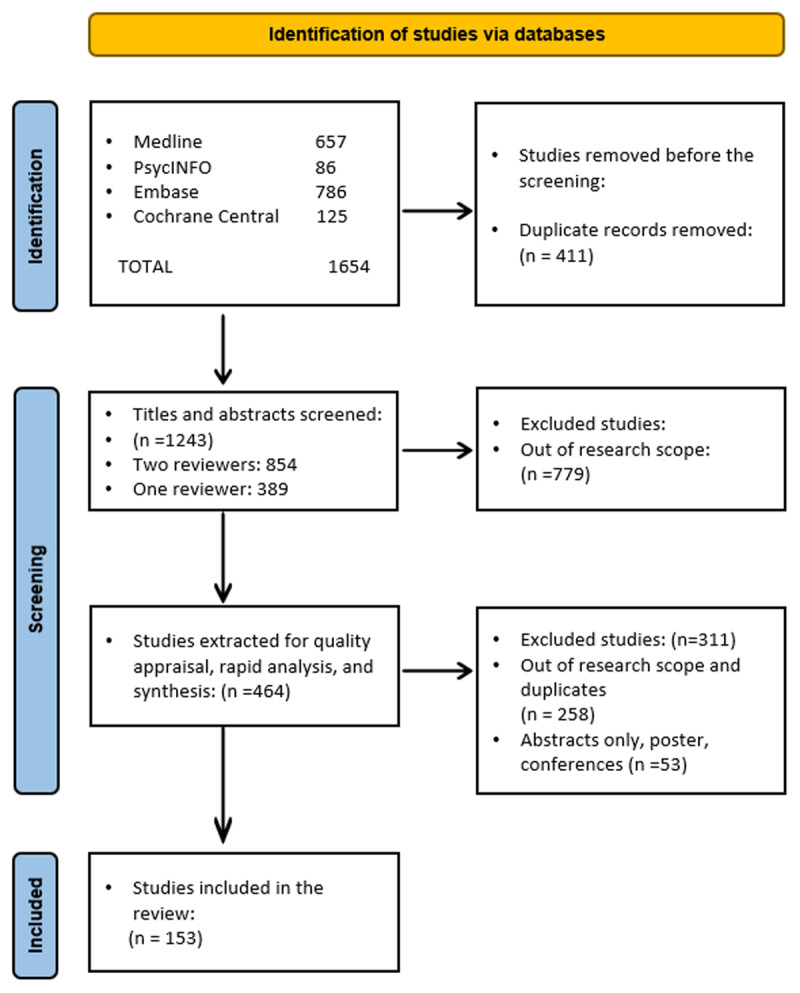
Adapted PRISMA flowchart [15] of the study inclusion process.

The search strategy was developed in collaboration with an information professional for Medline, including all specified keywords and index terms, and adapted for Embase, PsycINFO, and Cochrane CENTRAL [12]. As a rapid review, the search was limited to studies published in English from January 2010 until July 2022, considering Medical Subject Headings (MeSH): Chronic disease AND Continuity of care AND Indigenous AND Qualitative [12]. The flow of studies through the selection process is displayed in Figure 3.

### Participants

The review included studies involving First Nations Peoples from Australia, Canada, and Aotearoa, focusing on chronic conditions: cancer, cardiovascular disease, chronic kidney disease, diabetes, and their complications. It embraced the authors’ definitions of ‘First Nations’ without re-identification efforts [12].

### Context

Contextual considerations involved public health systems at individual, country, and aggregated levels in Australia, Canada, and Aotearoa. The review aggregated context data as “First Nations Peoples” while reporting each country’s specifics [12].

### Types of Studies

Qualitative study designs and qualitative aspects of mixed methods studies were included [12].

### Data Extraction

Data extraction covered population specifics, context, culture, location, study methods, chronic conditions, health system barriers and enablers following WHO’s IPCHS principles [4, 12].

### Data Synthesis

We assessed study quality using the JBI Critical Appraisal Checklist for Qualitative Research and The Aboriginal and Torres Strait Islander Quality Appraisal Tool [12]. Qualitative research findings were categorised based on themes from Figure 1 to classify barriers and Figure 2 to recognise enablers [4], sorted, coded, and presented in figures revealing frequencies to summarise themes [14]. Narrative interpretation was employed to group, illustrate, and explain findings, with results reviewed by all co-authors. Details can be found in the published protocol [12].

## Results

Of 1654 articles identified from databases, 153 studies were included (Figure 3).

### Characteristics of included studies

**Figure 4:**
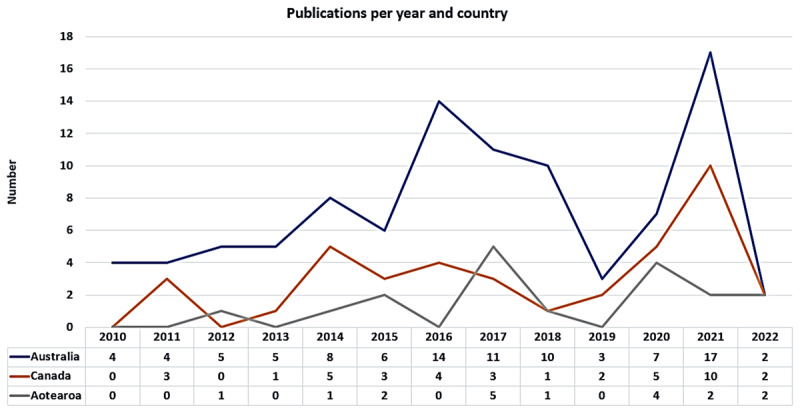
Characteristics of the included studies: country, number of publications and years.

Of the 153 studies included, 96 studies (63%) were from Australia with Aboriginal and Torres Strait Islander Peoples; Canada had 39 studies (25%), mainly with First Nations and Métis Peoples, and Inuit; and Aotearoa had 18 studies (12%) mostly with Māori people. Most studies were published between 2013 and 2021, as reflected in Figure 4.

### Chronic diseases

**Figure 5:**
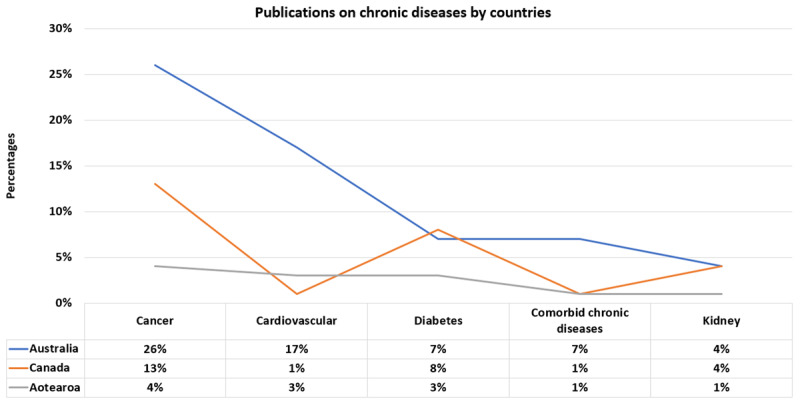
Included publications on chronic conditions by countries.

Forty-three per cent of the studies investigated cancer, 20% examined cardiovascular health (cardiovascular), 19% diabetes, 9% kidney disease, and another 9% referred to comorbid chronic diseases within the study scope (Appendix I) [16]. Australia had the highest percentage of cancer (26%) and cardiovascular health publications (17%), and Australia and Canada had a similar percentage of publications on diabetes and kidney disease. Aotearoa had the least number of studies except for cardiovascular health (Figure 5).

### Assessment of studies, methods and quality

**Figure 6:**
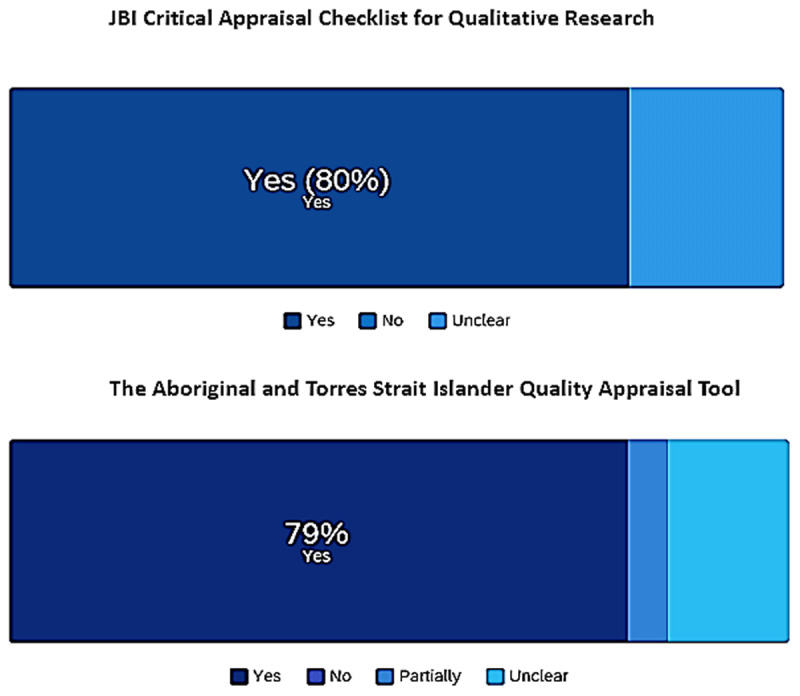
Summarised results from quality appraisal tools, where: yes, no, partially and unclear, refer to the studies meeting each tool’ summarised quality requirements or item responses.

More than 65% of papers used qualitative methods, and 13% referred to qualitative methods specifying indigenous practices such as Yarning, Hui, Fono, and storytelling. Approximately 22% of the studies used mixed methods. Two quality appraisal tools were utilised (A and B, see Appendix II [12, 16]. The summarised quality of the captured studies is aggregated in Figure 6, reflecting approximately 80% quality according to such tools.

#### The barriers to continuity of care

**Figure 7:**
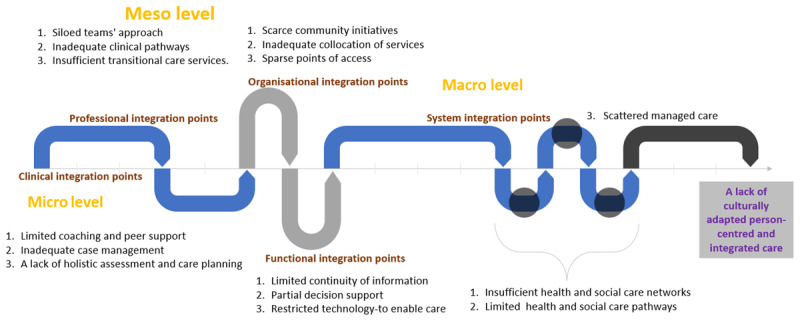
Main points across the health system at which continuity and care coordination barriers emerged for First Nations Peoples [17,18,19,20,21,22,23,24,25,26,27,28,29,30,31]: At the micro level and clinical integration points, [4] individuals face constraints such as limited access to coaching and peer support [28, 32, 33] and deficiencies in case management and holistic care planning [34,35,36,37,38,39]. The meso level presents challenges in terms of professional integration points [4], characterised by siloed team approaches [40, 41], inadequate clinical pathways [42, 43], and gaps in transitional care services [25, 44]. Organisational integration points [4] reveal barriers related to the absence of community initiatives [21, 45, 46], challenges in service collocation [47,48,49], and issues surrounding points of access [38, 50]. Functional integration points [4] present obstacles primarily in continuity of information [32, 39, 41], decision support [51, 52], and the utilisation of technology for care enablement [53, 54]. The macro level, focusing on system integration points [4], highlights significant limitations, including deficient health and social care networks [24, 55, 56], constrained health and social care pathways [57,58,59], and challenges related to care management [60,61,62]. For a more detailed analysis and synthesis of the studies underpinning Figure 7, please refer to Figure 8 and subsequent narrative explanations.

**Figure 8:**
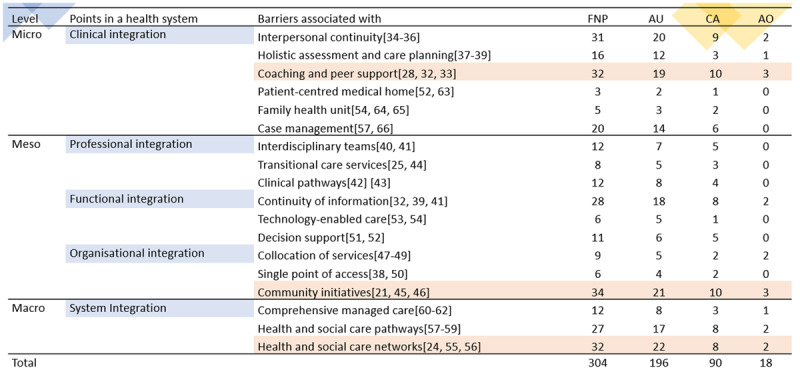
The points in a health system where barriers to continuity and coordination of care emerged for First Nations Peoples – The figure was created based on WHO’s IPSCH and is citing examples from reviewed literature; the numbers in columns represent the frequency of each WHO’s IPSCH theme as per reviewers’ cataloguing using Figure 1 as a guide [4]. The cells highlighted in orange represent the most frequent themes. In the columns, FNP refers to First Nations Peoples, AU to Australia, CA to Canada and AO to Aotearoa.

Barriers and enablers can describe the same issues in negative or positive contexts. We collected barrier data from 93 papers (Appendix III) [16] and assessed points within the health system where barriers to continuity and care coordination emerged for First Nations Peoples, guided by Figure 2 [4]. Figure 7 outlines the top three points identified across the health system where these barriers appeared in the literature. Figure 8 offers a comprehensive overview of the primary continuity and coordination of care barriers identified for each country and as a group across micro, meso, and macro levels (as defined by WHO’s IPSCH [4, 17,18,19,20,21,22,23,24,25,26,27,28,29,30,31].

The First Nations Peoples analysis (Figure 8) was sorted by barrier’s weight (frequency) to identify the three main barriers and provide a narrative analysis of key examples from the literature reviewed:

The most frequent barrier: community initiatives (Meso level – organisational integration): This refers to requiring community initiatives because of deficient culturally appropriate and person-centred approaches involving patients and family members [17, 18]. Studies on diabetes and kidney disease in Australia and Canada found culturally specific barriers in community screening, as people would prefer services provided by culturally competent healthcare professionals with support from the community and people with lived experience [17, 18]. Such community initiatives were necessary to deliver positive experiences on tertiary health services for First Nation Peoples [17, 18]. Similarly, a cardiovascular study identified that health professionals need more awareness of Aboriginal Peoples in Canada, particularly around their needs; therefore, it was recommended that clinicians get training on traditional practices and cultural competency to understand the communities they serve [20]. These barriers imply a lack of involvement of caregivers in care processes, cultural training for care workers, and deficient health workers’ education and awareness of the effects of colonisation on communities [19, 20]. Equally, a Canadian investigation of First Nations adolescents with type 2 diabetes suggests that not having community initiatives contributes to stigma and shame and limits the patient’s responses to lifestyle interventions and pharmacotherapy designs [21]. Cancer research uncovered that more personal control strategies for under-screened Canadian First Nations women are necessary (i.e., self-sampling in combination with community engagement and culturally sensitive education); this is reflected in the qualitative data from a randomised controlled trial, which found that such strategies brought less physical and emotional discomfort and fewer concerns regarding the privacy of test results [22]. According to other Canadian investigations by Nunavut partners on end-of-life service delivery for cancer, people would prefer receiving care within their communities (in their territories); they refer to wanting to pass away at home with their family involved in the process; this highlighted the challenges this population face outside of their communities (e.g., communication, extensive medical travel and lack of culturally appropriate care). Therefore, they valued service providers with strong ties to the community (their absence was a critical barrier) [23].

The second most frequent barrier: health and social care networks (Macro level – system integration): This denotes many types of connections; for instance, according to Māori perceptions, links between the patients and their whānau (families) and health providers; the absence of such relationships impedes the creation of networks and limits the trust and confidence in services that could facilitate opportunities for broader engagement with the community to promote health [24]. A study on First Nation Peoples’ chronic diseases and hospital readmission in Australia indicated that patients readmitted to hospitals experienced poor access to reliable community and social services because of housing deprivation, a lack of support from a carer or health professional for chronic disease self-management, inadequate discharge planning and poor community health follow up. These factors impacted their ability to manage their illnesses in the community because of a lack of social and care networks. Participants indicated that accessing transport services provided by Aboriginal health or community services was essential. Considering the lack of these services, researchers point out that further supporting health and social care is vital for addressing health disparity gaps around culturally appropriate benefits, disability and housing challenges [26]. Similarly, health networks were considered essential to receive cancer care for people living in remote areas in Canada; people were at risk of experiencing stress as they had to leave their family and community support to travel to receive care. The healthcare systems did not accommodate the context and logistic complexity of healthcare access and the increased risk of harm during their transition to urban centres [27]. Such care networks require employing First Nations Peoples [25], because, without adequate health and social care networks, there was a lack of communication between departments and/or hospitals [28]; for example, patients and general practitioners (GPs) in Aotearoa emphasised the need for coordinating collaboration with the whānau as the lack of such networks delayed earlier diagnosis and treatment of lung cancer. The size and importance of this barrier were so significant that GPs admitted to sending all referrals as having a high suspicion of lung cancer (incorrectly applying the criteria) to ensure prompt referral acceptance [28].

The third most frequent barrier: coaching and peer support (Micro level – clinical integration): This barrier seems to merge explanations from the previous two in this synthesis; it highlights the importance of resourcing and supporting community initiatives that strengthen health care and social networks via peer support and coaching. Not having these services affected people in many ways; for example, it exacerbated logistics costs and appointment adherence stress because receiving healthcare from clinicians/professionals without cultural competencies generated mistrust of the healthcare system and exposure to racism [29]. Correspondingly, a study on health service delivery for Aboriginal and Torres Strait Islander Peoples with chronic illness referred to poor access to culturally appropriate health services, dislocation from cultural support systems, poor communication with health care professionals, and racism [30]. Addressing impediments to the fundamental role of coaching and peer support in healthcare for First Nations Peoples required clinical integration and culturally responsive leadership [31]. Not having these leaders (peers or people from the community) meant lacking who spoke the local languages and held influence, respect, and connection within communities [31]. Such deficits in peer support services denied the powerful force of committed, caring, and passionate continuity of care grassroots approaches [31]. For instance, research that sought to understand how care coordination influences Aboriginal Peoples’ experiences of cancer treatment in Australia found that accessing services without peers or family to accompany patients during treatment impacted them negatively because of limited access to interpreters and other cultural brokers [29]. This insufficiency of cultural safety within hospitals generated shame in diagnosis and multiple stressors competing with the management of cancer treatment (i.e., finances, family needs, housing) [29]. These issues were credited partly to the lack of coaching and peer support in the form of staff or peers that can recognise cultural issues and solve them appropriately, e.g., by speaking Aboriginal languages and understanding the most effective cultural and feasible ways to facilitate better care [29].

#### The enablers to continuity of care

**Figure 9:**
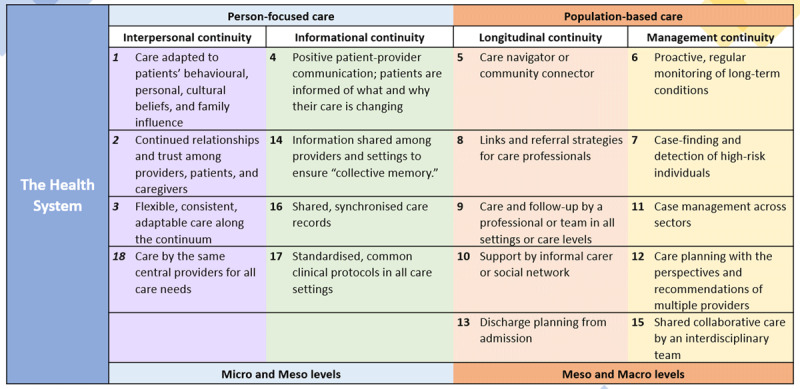
Decision-making matrix to co-design evidence-based care solutions with First Nations Peoples [16]: This matrix includes the most important enablers and strategies for achieving continuity of care and its integration for First Nations Peoples living with chronic conditions, as per the synthesis of 103 publications. It situates the reader on the order of importance of needed strategies (nature, levels, and types) around enabling care for First Nations Peoples [23, 40, 65, 67,68,69,70,71]. These enablers are ranked in order of importance with numbers from 1–18, considering the frequency of WHO’s IPSCH [4] theme identification within the qualitative review process.

Figure 9 highlights crucial enablers and strategies for achieving care continuity and integration among First Nations Peoples with chronic conditions [23, 40, 65, 67,68,69,70,71]. This evidence-based synthesis of 103 publications prioritises strategies into a decision-making matrix to co-design care solutions with First Nations Peoples [16], and its content is explained in a narrative format after.

As reflected in Figure 9 [16], the supported care pathway underscores the importance of tailoring care to individuals’ unique needs as they transition within the healthcare system; it involves receiving care adapted to them and their behavioural, personal, cultural beliefs and family influence [72,73,74]. A continued relationship and trust must further facilitate this care among providers, patients, and caregivers [75, 76]. Such a process requires flexible, consistent, adaptable care along the continuum [77, 78]. The services must be maintained by constant and consistent communication via an adjustable care plan that allows for creating and maintaining a relationship based on precise information and instructions about the agreed care strategy [42, 44, 55, 79, 80].

**Figure 10:**
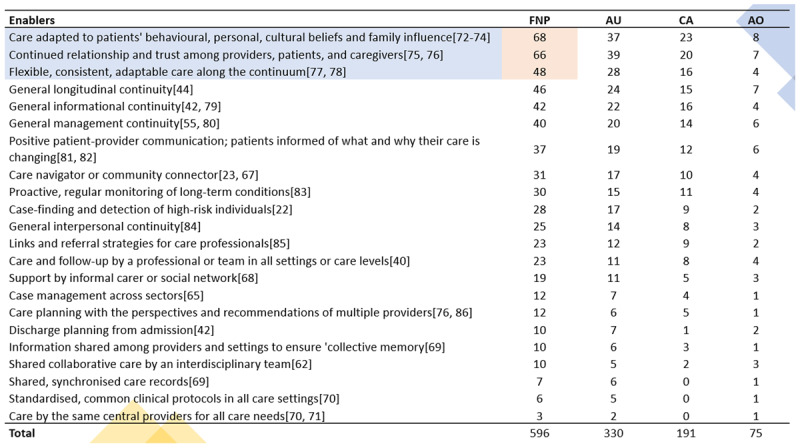
Enablers for achieving continuity of care for and with First Nations Peoples – The figure was created based on WHO’s IPSCH by citing examples from reviewed literature; the numbers in columns represent the frequency of each WHO’s IPSCH theme as per reviewers’ cataloguing using Figure 2 as a guide [4]. The cells highlighted in orange represent the most frequent themes. In the columns, FNP refers to First Nations Peoples, AU to Australia, CA to Canada and AO to Aotearoa.

In the enabled care process (Figure 9), the person and their family would be better positioned to understand how their care may be changed [81, 82] because a care navigator/community connector [23, 67] would be there to support the proactive and regular monitoring of their long-term conditions [23, 67, 83]. This connector encourages the detection of high-risk conditions of the person and their family members [22, 42, 62, 69, 76, 84,85,86] because connectors must be positioned within the community or and/or be peers or a part of the family or group (i.e., local population and/or located within the same geographical location of the community); thus, the connector facilitates the integration of services (e.g., links and referral strategies to other care professionals) and supports a shared understanding between informal cares and social care networks, facilitating care management across micro, meso and macro levels of the health system (see Figure 9 [16] and detailed analysis in Figure 10) [23, 40, 63, 65,66,67,68,69,70,71].

Figure 10 presents the range of approaches and interventions for achieving continuity of care (extracted and analysed using Figure 2 as a lens) [4]. It offers example citations from 103 publications (see Appendix VI) [16] that met the enablers of continuity of care criteria per reviewers’ assessment, organised in descending order of theme frequency.

Figure 10 reveals a pattern that coincides across countries. The top three enablers from this synthesis are explained and illustrated via insights from reviewed literature:

The most frequent enabler: care adapted to patients’ behavioural, personal, cultural beliefs, and family influence: This theme denotes a variety of factors related to the sociocultural determinants of health and being connected to community and culture, and having access to Aboriginal primary health care as a protective factor, e.g., the perspective of Aboriginal and Torres Strait Islander primary health care providers in the cancer field (well women’s screening), highlights the sensitivities to women’s emotional and cultural needs, leveraging on gender-specific community channels and understanding cultural beliefs which inform behaviours, e.g., flexibility by opening on Saturdays and extended weekday hours, having varying start times, and encouraging women to combine breast and cervical screening appointments [71]. The perspective on this enabler from an Australian (New South Wales-based) Elders group (Cancer) involved a connection to country and ancestors, increasing the feasibility of healthy behaviours, and addressing some of the barriers to healthcare access via strategies like transport subsidies and promoting awareness of such services where they are in place [87]. In kidney research, papers referred to modifying the care environments by creating a welcoming clinical space where families could visit and be accommodated [88]. This modification required improved cultural competency from staff and an increased understanding of the impacts for people being off-country and away from their communities, implementing patient-led coordinated care, including sustaining an Indigenous Patient Reference Group to support ongoing healthcare service and decision processes [88]. The study recognised these groups’ socioeconomic limitations and suggested logistics support like transport to clinics and accommodation [88]. Likewise, an Australian study identified participants requiring cultural and traditional knowledge and insights from their own experiences [30]. Participants said that while they often felt exhausted and bewildered by the burden of chronic illness, they drew strength from being connected to their Aboriginal community by having regular and ongoing access to primary health care and being a part of a supportive family network. Within this context, Elders played an essential role in increasing people’s awareness of the impact of chronic illness on individuals and communities [30]. Comparably, an Aotearoa study found that involving community leaders (e.g., models in sports, music, and comedy) could help adapt care and challenge the status quo by involving them in formal policy, health, and governance [31].

The second most frequent enabler: a continued relationship and trust among providers, patients, and caregivers: According to cardiovascular research [89], this enabler reflects developing a trusting relationship and making people feel comfortable and safe within the health service environment, particularly for the First Nations Peoples, as they wanted to feel free from judgment and to be treated with respect (preventing historical treatment and negative experiences within the healthcare system) [89]. Using a mixed methods approach, a cardiac rehabilitation investigation found that increasing health professionals’ knowledge, skills, and confidence in working with First Nations Peoples improved attendance when co-designing the intervention [89]. Similar findings from the perspective of Inuit, First Nations, and Métis Peoples were identified in cancer research [78]. These studies found that knowledge translation methods were necessary to create coaching/shared decision-making approaches, essential for building trusting relationships and safer care services [78]. For instance, for women’s cancer screening in Australia (Yolngu women) [25], it was necessary to promote community role models at social information sessions to encourage awareness and attendance to early detection services [25]. These activities involved using local social places and providing food to facilitate a welcoming and trusting environment and relationships to discuss sensitive health issues (considered women’s businesses in their care management). For enabling such relationships and trust-creation processes, humour was fundamental to strengthening health and social connections because, according to the study, laughing was a good connection strategy for Yolngu women [25].

The third most frequent enabler: patients and caregivers providing flexible, consistent, adaptable care along the continuum: Research evaluation on First Nations patient experiences around kidney disease demonstrated that flexible arrangements of appropriate transport and accommodation were enabled by establishing good communication with patients, particularly those from remote communities, by advertising trips [75]. The study was based on a dialysis bus (which visited remote communities for one to two weeks for people to receive their dialysis in their own territories (country) [75]. It involved creating the role of ‘a champion of the bus’: a coordinator who liaised with communities continually to ensure they were prepared for trips. The process was centred on First Peoples’ local knowledge (cultural and practical); for example, knowledge of the country (lands and roads) and how to use the bus as a health promotional tool for kidney disease prevention for families and patients [75]. Similar examples in Canada adapted care along the continuum via technology and a model developed by First Nations communities and OKAKI Health Intelligence Inc.: In the RADAR, ‘Reorganising the Approach to Diabetes care through the Application of Registries’, all communities had representation (remote care coordinators and registered nurses supported local healthcare providers in First Nations communities through telehealth) [90]. The RADAR has a steering committee to guide the project. Within such a remote-support model, they used a shared electronic health record/diabetes registry called CARE, which contains clinical patient data on key diabetes outcomes and quality of care indicators populated and maintained by local healthcare providers and remote care coordinators. They directed population-level care (to identify gaps, recommend and/or implement therapeutic changes) and coordinated referrals using current clinical practice guidelines through regular case review and conferencing [90].

## Discussion

Despite differences across countries, First Nations Peoples, diseases, and approaches, we have identified general trends across enablers and barriers to continuity of care. The analysis demonstrates high consistency across countries around improving and developing further health and social care networks, community initiatives, interpersonal continuity, coaching and peer support. The pattern is reflected as a call for supporting and strengthening community connections and harmonising Western healthcare practices with health and well-being practices from First Nations Peoples. The evidence indicates that employing local First Nations Peoples in care designs and delivery across the care spectrum may substantially improve health, socioeconomic and communal life [49, 91]. A pressing need for tailoring culturally responsive services, including biomedical and clinical care provision, was identified across all levels of the care continuum within health systems [60]. The most required action involves supporting the co-design of adequately resourced networks [92] with each community [18]; while considering the effects of enduring colonisation, including but not limited to the systematic dismantling of First Nations societies and kinship structures, disconnection from country, dispossession of language and cultural practices and systemic racism and discrimination [66, 93, 94].

Since focusing only on barriers to reducing inequities in healthcare outcomes opens mainly deficit-based discussions that isolate global socioeconomic, political, contextual, and infrastructure disparities experienced by First Nations Peoples, it was appropriate to broaden the analysis towards barriers and enablers with consideration beyond the biomedical model, towards psychosocial approaches [4, 95, 96]. To that end, our review demonstrates that enablers and barriers to continuity of care and care integration for First Nations Peoples are a continuum. The difficulties in detangling these factors and components (i.e., barriers and enablers) from each other are typical of complex or ‘wicked’ problems [64], as they refer to diverse interactions (knowledge translation processes) between people, services, governments, institutions, and the power and dominance of Western paradigms over First Nations paradigms, a result of colonisation [97]. Yet, the traditional problem-solving methods [57] of First Nations Peoples are proven through survival across millennia of lived experiences of countless generations. Therefore, the results of the review point towards incorporating First Nations Peoples’ collaborative and participatory methods, which Western systems have only recently begun to recognise [98,99,100]. Currently, the importance of working in partnership with groups/communities and relevant stakeholders via dialogue [101] and mixed methods co-design/evaluation is better understood and deemed necessary [4, 95,96,97,98,99,100].

Using lenses from the WHO’s IPSCH [4], this review identified culturally responsive person-centred and integrated care means for consciously delivering care by incorporating individuals, carers, families, and the community’s perspectives around their comprehensive needs rather than their diseases. Repeated trends across findings call for respecting social preferences and supporting First Nation Peoples to participate in care environments and decisions, specifically via culturally responsive person-centred and integrated care that encompasses all levels of health systems, from the individual clinical encounter to the formulation of health policy and associated health and wellbeing services and systems [4, 22, 23, 40, 42, 62, 65, 67,68,69,70,71, 76, 84,85,86]. Our synthesis implies that reforming services and systems must involve all stakeholders to support the safe, appropriate, and readily available strategies that facilitate the optimal care journey for First Nations Peoples; this requires constant connection and assessment of health and social care networks across systems [76, 98,99,100, 102, 103].

A recent review highlights limited evidence regarding cost-effective investments in Aboriginal and Torres Strait Islander healthcare [104]. However, the cost to the health system of potentially preventable hospitalisations for chronic conditions for Aboriginal and Torres Strait Islander people has been demonstrated [1, 105,106,107]. Our findings strongly support the need for governments to consider embracing social return on investment to understand and enhance the well-being of First Nations Peoples, capitalising on its health spending per person [104], to address substantial health disparities as observed in Aotearoa [108]. These propositions are supported by evidence showing how to mitigate disparities by strengthening primary healthcare [107, 109]. Recognising diverse health perspectives considering individual, familial, and cultural differences is crucial for avoiding a one-size-fits-all approach. The concept of “local biologies” has emphasised this, contemplating how various social and physical conditions impact health. A prime illustration of this can be seen in various healthcare studies that highlight the significance of tailored healthcare strategies for addressing drivers of disparities [69, 98,99,100, 110].

In recognising First Nations Peoples knowledges of health and wellbeing as the spiritual, social, emotional and physical health and wellbeing of the collective across generations, a co-creation approach is fundamental in designing responsive care [76, 98,99,100, 102, 103, 111]. For example, care navigators or community connectors who support journeying between two worlds and provide continuity can enable healthcare services to respond to these knowledges (longitudinal continuity). Effective partnerships between healthcare professionals and First Nations communities across care settings (management continuity) are essential in this process. These recommendations align with similar Australian national-level studies on healthcare and implementation science [69, 98,99,100, 111] and resonate with the experiences of First Nations Peoples in the South American context [112]. These navigators and community connectors actively bridge healthcare realms while respecting and preserving First Nations Peoples’ worldviews and knowledges [69, 98,99,100, 110, 112].

## Limitations and strengths

With the nature of a rapid review, limited to research published from January 2010 to July 2022, the possibility of missing relevant insights from multimethod studies is acknowledged. Restricting the search only to qualitative findings published in English may have excluded meaningful experiences from unpublished research, quantitative analyses, conferences, talks, posters, reports, and non-academic, non-English publications. Additionally, using a strict lensing format or categorisation may have influenced the grouping of themes in ways that constrict a broader meaning of the data (particularly data produced by First Nations Peoples) and its interpretation. This review will inform the development of an evidence-informed protocol for continuity of care for Aboriginal and Torres Strait Islander Peoples living with chronic conditions [7, 8]. Its practice focus brings insight ready to activate change involving patients, health practitioners, policymakers, organised communities, and advocates. The evidence-based decision-making matrix provides a template for co-designing care solutions with First Nations Peoples using participatory action research strategies [4, 16, 97, 113,114,115]. This practical approach can facilitate change towards a more appropriate, accessible, acceptable, culturally safe, and quality-coordinated focus [4, 113,114,115,116].

## Conclusion

Health and social care should be harmonised with First Nations Peoples’ cultural beliefs and family influences. Sustainable strategies require a co-design commitment for well-funded flexible care plans considering coaching and peer support across the lifespan. Our synthesis delivers an evidence-based decision-making matrix to support the co-design of care solutions with First Nations Peoples [16]. These findings could aid clinicians, researchers, politicians, and community advocates to collaborate with First Nations Peoples and co-develop, implement, and evaluate better continuity of care for First Nations Peoples living with chronic conditions.

## References

[1] Banham D, Chen T, Karnon J, Brown A, Lynch J. Sociodemographic variations in the amount, duration and cost of potentially preventable hospitalisation for chronic conditions among Aboriginal and non-Aboriginal Australians: a period prevalence study of linked public hospital data. BMJ open. 2017; 7 e017331. DOI: 10.1136/bmjopen-2017-017331 PMC5652548 29038183

[2] AIHW. 2022 Australian Burden of Disease Study. Interactive data on risk factor burden among Aboriginal and Torres Strait Islander people. (Canberra: Australian Institute of Health and Welfare); 2018.

[3] AIHW. Disparities in potentially preventable hospitalisations across Australia, 2012–13 to 2017–18. (Canberra: AIHW); 2020.

[4] WHO. Continuity and coordination of care: a practice brief to support implementation of the WHO Framework on integrated people-centred health services; 2018.

[5] Valentijn PP, Schepman SM, Opheij W, Bruijnzeels MA. Understanding integrated care: a comprehensive conceptual framework based on the integrative functions of primary care. International journal of integrated care. 2013; 13. DOI: 10.5334/ijic.886 PMC3653278 23687482

[6] de Witt A, Matthews V, Bailie R, Garvey G, Valery P C, Adams J, Martin JH, Cunningham, FC. Communication, Collaboration and Care Coordination: The Three-Point Guide to Cancer Care Provision for Aboriginal and Torres Strait Islander Australians. International Journal of Integrated Care [Electronic Resource]. 2020; 20: 10. DOI: 10.5334/ijic.5456 PMC7292184 32565760

[7] Brown A, Keech W, McBride K, Kelly J, Stewart H, Dowling A, Wall J. The South Australian Aboriginal Heart and Stroke Gap Analysis for the South Australian Aboriginal Heart and Stroke Plan 2017–2021; 2016.

[8] SAACC. SA Aboriginal Chronic Disease Consortium Road Map for Action 2017–2021. ed S A A C D Consortium (The Consortium: South Australian Aboriginal Chronic Disease Consortium); 2017–2021.

[9] Glover M, Kira A, Johnston V, Walker N, Thomas D, Chang AB, Bullen C, Segan C, Brown N. A systematic review of barriers and facilitators to participation in randomized controlled trials by Indigenous people from New Zealand, Australia, Canada and the United States. Global Health Promotion. 2015; 22: 21–31. DOI: 10.1177/1757975914528961 24842989

[10] Redwood S, Gill PS. Under-representation of minority ethnic groups in research — call for action. British Journal of General Practice. 2013; 63: 342–3. DOI: 10.3399/bjgp13X668456 PMC3693777 23834862

[11] King M, Smith A, Gracey M. Indigenous health part 2: the underlying causes of the health gap. The Lancet. 2009; 374: 76–85. DOI: 10.1016/S0140-6736(09)60827-8 19577696

[12] Pinero de Plaza MA, Brown S, Wu C-J, Robyn C, McBride K, Gebremichael L, Pearson O, Hines S, Morey K. System enablers and barriers to continuity of care for First Nations people living with chronic conditions: A rapid qualitative review protocol. figshare; 2022. 10.5334/ijic.7643 PMC10723014 38107834

[13] Garritty C, Gartlehner G, Nussbaumer-Streit B, King VJ, Hamel C, Kamel C, Affengruber L, Stevens A. Cochrane Rapid Reviews Methods Group offers evidence-informed guidance to conduct rapid reviews. Journal of clinical epidemiology. 2021; 130: 13–22. DOI: 10.1016/j.jclinepi.2020.10.007 33068715 PMC7557165

[14] Griffiths TH. Application of summative content analysis to a postal questionnaire. Nurse Researcher. 2016; 23. DOI: 10.7748/nr.23.3.30.s7 26793985

[15] Page MJ, Moher D, Bossuyt PM, Boutron I, Hoffmann TC, Mulrow CD, Shamseer L, Tetzlaff JM, Akl EA, Brennan SE. PRISMA 2020 explanation and elaboration: updated guidance and exemplars for reporting systematic reviews. bmj. 2021; 372. DOI: 10.1136/bmj.n160 PMC8005925 33781993

[16] Pinero de Plaza MA. Decision-Making Matrix to Co-design Evidence-based Care Solutions with First Nations Peoples. Flinders University; 2023. DOI: 10.25451/flinders.24104940.v1

[17] Taylor EV, Lyford M, Holloway M, Parsons L, Mason T, Sabesan S, Thompson SC. “The support has been brilliant”: experiences of Aboriginal and Torres Strait Islander patients attending two high performing cancer services. BMC Health Services Research. 2021; 21. DOI: 10.1186/s12913-021-06535-9 PMC8142293 34030670

[18] Blair T, Babyn P, Kewistep G, Kappel J, Stryker R, Ramsden VR, Neudorf C, Levandoski C. Program Report: *Nîsohkamâtowak*—Helping Patients and Families Living With Kidney Disease in Northern Saskatchewan. Canadian Journal of Kidney Health and Disease. 2022; 9: 205435812110670. DOI: 10.1177/20543581211067071 PMC8753229 35035983

[19] Maiorana ACE, Venables M, Dubrawski K, Dowling T, et al. Reducing inequities in aboriginal Australian heart health through culturally specific cardiac rehabilitation. 2015; 22(1): 15.

[20] DiGiacomo ML, Thompson SC, Smith JS, Taylor KP, Dimer LA, Ali MA, Wood MM, Leahy TG, Davidson PM. ‘I don’t know why they don’t come’: barriers to participation in cardiac rehabilitation. Australian Health Review. 2010; 34: 452–7. DOI: 10.1071/AH09803 21108907

[21] Wicklow B, Dart A, McKee J, Griffiths A, Malik S, Quoquat S, Bruce S. Experiences of First Nations adolescents living with type 2 diabetes: a focus group study. Canadian Medical Association Journal. 2021; 193: E403–E9. DOI: 10.1503/cmaj.201685 33753364 PMC8096390

[22] Zehbe I, Wakewich P, King A-D, Morrisseau K, Tuck C. Self-administered versus provider-directed sampling in the Anishinaabek Cervical Cancer Screening Study (ACCSS): a qualitative investigation with Canadian First Nations women. BMJ Open. 2017; 7: e017384. DOI: 10.1136/bmjopen-2017-017384 PMC5588934 28864487

[23] Galloway T, Horlick S, Cherba M, Cole M, Woodgate RL Healey Akearok G. Perspectives of Nunavut patients and families on their cancer and end of life care experiences. International Journal of Circumpolar Health. 2020; 79: 1766319. DOI: 10.1080/22423982.2020.1766319 32449489 PMC7448904

[24] Slater T, Matheson A, Ellison-Loschmann L, Davies C, Earp R, Gellatly K, Holdaway M. Exploring Māori cancer patients’, their families’, community and hospice views of hospice care. International journal of palliative nursing. 2015; 21: 439–45. DOI: 10.12968/ijpn.2015.21.9.439 26412274

[25] McGrath P, Rawson N. Key factors impacting on diagnosis and treatment for vulvar cancer for Indigenous women: Findings from Australia Supportive Care in Cancer. 2013; 21: 2769–75. DOI: 10.1007/s00520-013-1859-7 23720063

[26] Jayakody A, Carey M, Bryant J, Ella S, Hussein P, Warren E, Bacon S, Field B, Sanson-Fisher R. Exploring experiences and perceptions of Aboriginal and Torres Strait Islander peoples readmitted to hospital with chronic disease in New South Wales, Australia: a qualitative study. Australian Health Review. 2021; 45: 411–7. DOI: 10.1071/AH20342 34334156

[27] Jull J, Sheppard AJ, Hizaka A, Barton G, Doering P, Dorschner D, Edgecombe N, Ellis M, Graham ID, Habash M, Jodouin G, Kilabuk L, Koonoo T, Roberts C. Experiences of Inuit in Canada who travel from remote settings for cancer care and impacts on decision making. BMC Health Services Research. 2021; 21. DOI: 10.1186/s12913-021-06303-9 PMC8042963 33845810

[28] Kidd J, Cassim S, Rolleston A, Chepulis L, Hokowhitu B, Keenan R, Wong J, Firth M, Middleton K, Aitken D, Lawrenson R. Hā Ora: secondary care barriers and enablers to early diagnosis of lung cancer for Māori communities. BMC Cancer. 2021; 21. DOI: 10.1186/s12885-021-07862-0 PMC7863263 33541294

[29] Reilly R, Micklem J, Yerrell P, Banham D, Morey K, Stajic J, Eckert M, Lawrence M, Stewart HB, Brown A. Aboriginal experiences of cancer and care coordination: Lessons from the Cancer Data and Aboriginal Disparities (CanDAD) narratives. Health Expectations. 2018; 21: 927–36. DOI: 10.1111/hex.12687 29691974 PMC6186541

[30] Aspin C, Brown N, Jowsey T, Yen L, Leeder S. Strategic approaches to enhanced health service delivery for Aboriginal and Torres Strait Islander people with chronic illness: a qualitative study. BMC health services research. 2012; 12: 1–9. DOI: 10.1186/1472-6963-12-143 22682035 PMC3405462

[31] The National Hauora coalition, Anderson A, Brown R, Wheeler J, Jansen RM. Pacific Fono: a community-based initiative to improve rheumatic fever service delivery for Pacific Peoples in South Auckland. Journal of Primary Health Care. 2020; 12: 384. DOI: 10.1071/HC20022 33349328

[32] Quinn E, O’Hara BJ, Ahmed N, Winch S, McGill B, Banovic D, Maxwell M, Rissel C. Enhancing the get healthy information and coaching service for Aboriginal adults: evaluation of the process and impact of the program. International Journal for Equity in Health. 2017; 16: 168. DOI: 10.1186/s12939-017-0641-8 28877697 PMC5586001

[33] Jull J, Sheppard AJ, Hizaka A, Barton G, Doering P, Dorschner D, Edgecombe N, Ellis M, Graham ID, Habash M, Jodouin G, Kilabuk L, Koonoo T, Roberts C. Experiences of Inuit in Canada who travel from remote settings for cancer care and impacts on decision making. BMC health services research. 2021; 21(1): 328. DOI: 10.1186/s12913-021-06303-9 33845810 PMC8042963

[34] Mooi JK, Whop LJ, Valery PC, Sabesan SS. Teleoncology for indigenous patients: the responses of patients and health workers. Australian Journal of Rural Health. 2012; 20: 265–9. DOI: 10.1111/j.1440-1584.2012.01302.x 22998201

[35] Santella C, Tratt E, Nyamiaka J, Whiteley Tukkiapik L, Styffe C, Gamelin R, Macdonald ME, Brassard P. Perceptions of Inuit Women and Non-Inuit Healthcare Providers on the Implementation of Human Papillomavirus Self-Sampling as an Alternative Cervical Cancer Screening Method in Nunavik, Northern Quebec. Qualitative Health Research. 2022; 10497323221090805. DOI: 10.1177/10497323221090805 35621363

[36] Pitama S, Cave T, Huria T, Lacey C, Cuddy J, Frizelle F. Exploring Maori health worker perspectives on colorectal cancer and screening. New Zealand Medical Journal. 2012; 125: 75–84. 22729062

[37] Anderson K, Devitt J, Cunningham J, Preece C, Jardine M, Cass A. If you can’t comply with dialysis, how do you expect me to trust you with transplantation? Australian nephrologists’ views on indigenous Australians’ ‘non-compliance’ and their suitability for kidney transplantation. International Journal for Equity in Health. 2012; 11(1): (no pagination). DOI: 10.1186/1475-9276-11-21 PMC3352022 22513223

[38] Arthur HM, Suskin N, Bayley M, Fortin M, Howlett J, Heckman G, Lewanczuk R. The Canadian Heart Health Strategy and Action Plan: Cardiac rehabilitation as an exemplar of chronic disease management. Canadian Journal of Cardiology. 2010; 26: 37–41. DOI: 10.1016/S0828-282X(10)70336-6 20101356 PMC2827223

[39] Walker RC, Abel S, Palmer SC, Walker C, Heays N, Tipene-Leach D. “We Need a System that’s Not Designed to Fail Maori”: Experiences of Racism Related to Kidney Transplantation in Aotearoa New Zealand. Journal of Racial & Ethnic Health Disparities. 2022; 11: 11. DOI: 10.21203/rs.3.rs-934210/v1 PMC8751454 35018578

[40] Gador-Whyte AP, Wakerman J, Campbell D, Lenthall S, Struber J, Hope A, Watson C. Cost of best-practice primary care management of chronic disease in a remote Aboriginal community. Medical Journal of Australia. 2014; 200: 663–6. DOI: 10.5694/mja13.11183 24938349

[41] Wood B, Burchell AN, Escott N, Little J, Maar M, Ogilvie G, Severini A, Bishop L, Morrisseau K, Zehbe I. Using community engagement to inform and implement a community-randomized controlled trial in the Anishinaabek cervical cancer screening study. Frontiers in Oncology. 2014 MAR; 4: (no pagination). DOI: 10.3389/fonc.2014.00027 PMC3928568 24600584

[42] Anderson K, Cunningham J, Devitt J, Preece C, Cass A. “Looking back to my family”: indigenous Australian patients’ experience of hemodialysis. BMC Nephrology. 2012; 13: 114. DOI: 10.1186/1471-2369-13-114 22992225 PMC3518174

[43] Arora S, Kurji AK, Tennant MT. Dismantling sociocultural barriers to eye care with tele-ophthalmology: lessons from an Alberta Cree community. Clinical & Investigative Medicine – Medecine Clinique et Experimentale. 2013; 36: E57–63. DOI: 10.25011/cim.v36i2.19567 23544606

[44] Coombes J, Hunter K, Mackean T, Holland AJA, Sullivan E, Ivers R. Factors that impact access to ongoing health care for First Nation children with a chronic condition. BMC Health Services Research. 2018; 18: 448. DOI: 10.1186/s12913-018-3263-y 29898727 PMC6001071

[45] Shahid S, Finn L, Bessarab D, Thompson SC. ‘Nowhere to room … nobody told them’: logistical and cultural impediments to Aboriginal peoples’ participation in cancer treatment. Australian Health Review. 2011; 35: 235–41. DOI: 10.1071/AH09835 21612740

[46] Ciccone N, Armstrong E, Adams M, Bessarab D, Hersh D, McAllister M, Godecke E, Coffin J. Yarning together: Developing a culturally secure rehabilitation approach for Aboriginal Australians after brain injury. Brain Impairment. 2019; 20(3): 313.

[47] Ristevski E, Thompson S, Kingaby S, Nightingale C, Iddawela M. Understanding Aboriginal Peoples’ Cultural and Family Connections Can Help Inform the Development of Culturally Appropriate Cancer Survivorship Models of Care. JCO Global Oncology. 2020; 6: 124–32. DOI: 10.1200/JGO.19.00109 32031446 PMC6998014

[48] Letendre A, Garvey G, King A, King M, Crowshoe R, Bill L, Caron NR, Elias B. Creating a Canadian Indigenous Research Network Against Cancer to Address Indigenous Cancer Disparities. JCO Global Oncology. 2020; 6: 92–8. DOI: 10.1200/JGO.19.00049 32031447 PMC6998023

[49] Anderson A, Mills C, Eggleton K. Whanau perceptions and experiences of acute rheumatic fever diagnosis for Maori in Northland, New Zealand. New Zealand Medical Journal. 2017; 130(1465): 80–8. 29121626

[50] Smith G, Kirkham R, Gunabarra C, Bokmakarray V, Burgess CP. ‘We can work together, talk together’: an Aboriginal Health Care Home. Australian Health Review. 2019; 43: 486–91. DOI: 10.1071/AH18107 30355439

[51] Thompson SC, Shahid S, Bessarab D, Durey A, Davidson PM. Not just bricks and mortar: planning hospital cancer services for Aboriginal people. BMC Research Notes. 2011; 4: 62. DOI: 10.1186/1756-0500-4-62 21401923 PMC3068108

[52] Naqshbandi Hayward M, Paquette-Warren J, Harris SB, Tyler M, Chirila A, Harris S, Reichert S, Thind A, Wylie L, Zwarenstein M, Hayward MN, Mequanint S, Tompkins J, Webster-Bogaert S, Hiwi BT, Zaran H, Esler J, Fournie M, McLellan J, Orcutt M, Barre E, Walsh A, Bhattacharyya O, Dannenbaum D, Dawson K, Wortman J, Dyck R, Episkenew JA, Green M, Hanley A, Parry M, Lavallee B, Macaulay A, Salsberg J, McComber A, Tobe S, Toth E, Bailie R, Collins K, de Oliveira C, Hindmarsh M, Rac V, Stanley L, Lewis J, Nose M, Parent B, Sundquist S, Houle L, Houle A, Montour-Lazare D, Emond J, Jacobs J, Littlechild R, Graham B, Littlechild T, Guy D, Onespot C, McComb IK, Dufour E, Jolly V, Diamond C, Jones J, Hadden D, DeYaeger A, O’Keefe T, Roberts A, Organ M, Keesickquayash P, Panacheese D, Kirkness S, Jebb M, Constant C, Deleary A, Nawash R, Sinclair L, McDonald H, Nickel B. Developing community-driven quality improvement initiatives to enhance chronic disease care in Indigenous communities in Canada: The FORGE AHEAD program protocol. Health Research Policy and Systems. 2016; 14(1) (no pagination). DOI: 10.1186/s12961-016-0127-y PMC4960663 27456349

[53] Puszka S, Dingwall KM, Sweet M, Nagel T. E-Mental Health Innovations for Aboriginal and Torres Strait Islander Australians: A Qualitative Study of Implementation Needs in Health Services. JMIR Mental Health. 2016; 3: e43. DOI: 10.2196/mental.5837 27644259 PMC5048059

[54] Barsky J, Hunter R, McAllister C, Yeates K, Campbell N, Liu P, Perkins N, Hua-Stewart D, Maar MA, Tobe SW. Analysis of the Implementation, User Perspectives, and Feedback From a Mobile Health Intervention for Individuals Living With Hypertension (DREAM-GLOBAL): Mixed Methods Study. JMIR MHealth and UHealth. 2019; 7: e12639. DOI: 10.2196/12639 31815678 PMC6928701

[55] Brown A. Acute coronary syndromes in indigenous Australians: opportunities for improving outcomes across the continuum of care Heart. Lung & Circulation. 2010; 19: 325–36. DOI: 10.1016/j.hlc.2010.02.011 20363187

[56] Pace R, Hiwi BT, Zaran H, Harris S. 24 – Implementation and Evaluation of a Diabetes Registry in First Nations Communities in Canada: Data From the FORGE AHEAD Study. Canadian Journal of Diabetes. 2020; 44(7 Supplement): S11. DOI: 10.1016/j.jcjd.2020.08.029

[57] Aspin C, Brown N, Jowsey T, Yen L, Leeder S. Strategic approaches to enhanced health service delivery for Aboriginal and Torres Strait Islander people with chronic illness: a qualitative study. BMC Health Services Research. 2012; 12: 143. DOI: 10.1186/1472-6963-12-143 22682035 PMC3405462

[58] MacKay D, Kirkham R, Freeman N, Murtha K, Van Dokkum P, Boyle J, Campbell S, Barzi F, Connors C, O’Dea K, Oats J, Zimmet P, Wenitong M, Sinha A, Hanley A J, Moore E, Peiris D, McLean A, Davis B, Whitbread C, McIntyre H D, Mein J, McDermott R, Corpus S, Canuto K, Shaw JE, Brown A, Maple-Brown L, Diabetes Across the Lifecourse: Northern Australia P. Improving systems of care during and after a pregnancy complicated by hyperglycaemia: A protocol for a complex health systems intervention. BMC Health Services Research. 2020; 20: 814. DOI: 10.1186/s12913-020-05680-x 32867837 PMC7461356

[59] Reid J, Koopu P, Burkhardt N, Stewart T, Anderson A, Harwood M. Oral and dental health and health care for Maori with type 2 diabetes: A qualitative study. Community Dentistry & Oral Epidemiology. 2020; 48: 101–8. DOI: 10.1111/cdoe.12501 31657040

[60] Thompson SC, Shahid S, DiGiacomo M, Pilkington L, Davidson PM. Making progress: the role of cancer councils in Australia in indigenous cancer control. BMC Public Health. 2014; 14: 347. DOI: 10.1186/1471-2458-14-347 24725974 PMC4004461

[61] Macdonald B, Engel K, Climenhaga D, Compton K, Quewezance S, Langley S. Diabetes care and management in Saskatchewan first nations: A province-wide approach. Canadian Journal of Diabetes. 2013; 2: S232–S3. DOI: 10.1016/j.jcjd.2013.03.397

[62] Kidd J, Cassim S, Rolleston A, Chepulis L, Hokowhitu B, Keenan R, Wong J, Firth M, Middleton K, Aitken D, Lawrenson R. Ha Ora: secondary care barriers and enablers to early diagnosis of lung cancer for Maori communities. BMC Cancer. 2021; 21: 121. DOI: 10.1186/s12885-021-07862-0 33541294 PMC7863263

[63] Sabesan S, Larkins S, Evans R, Varma S, Andrews A, Beuttner P, Brennan S, Young M. Telemedicine for rural cancer care in North Queensland: bringing cancer care home. Australian Journal of Rural Health. 2012; 20: 259–64. DOI: 10.1111/j.1440-1584.2012.01299.x 22998200

[64] Micklem J, Reilly R, Stewart H, Miller S, Yerrell P, McDonald F, Patterson P, Walker R, Morey K, Brown A. Supportive care for aboriginal youth affected by cancer: Building understanding from narratives to improve service provision. Supportive Care in Cancer. 2016; 24(1 Supplement 1): S106.

[65] Askew DA, Togni SJ, Schluter PJ, Rogers L, Egert S, Potter N, Hayman NE, Cass A, Brown AD. Investigating the feasibility, acceptability and appropriateness of outreach case management in an urban Aboriginal and Torres Strait Islander primary health care service: a mixed methods exploratory study. BMC health services research. 2016; 16: 178. DOI: 10.1186/s12913-016-1428-0 27175475 PMC4866273

[66] Cooper R, Pollock NJ, Affleck Z, Bain L, Hansen NL, Robertson K, Chatwood S. Patient healthcare experiences in the Northwest Territories, Canada: an analysis of news media articles. International Journal of Circumpolar Health. 2021; 80: 1886798. DOI: 10.1080/22423982.2021.1886798 33734041 PMC8725720

[67] Brown C, Shahid S, Bernardes CM, Toombs M, Clark PJ, Powell EE, Valery PC. Partnering with support persons and clinicians to improve the health care experiences of patients with cirrhosis. Journal of Clinical Nursing. 2022; 21: 21. 10.1111/jocn.16302 35451073

[68] Merritt SM, Holloway C, Boltong A. Havin’ a yarn: A project to support aboriginal people affected by cancer. Asia-Pacific Journal of Clinical Oncology. 2014; 8: 203.

[69] Bailie J, Laycock A, Matthews V, Bailie R. System-Level Action Required for Wide-Scale Improvement in Quality of Primary Health Care: Synthesis of Feedback from an Interactive Process to Promote Dissemination and Use of Aggregated Quality of Care Data. Frontiers in Public Health. 2016; 4: 86. DOI: 10.3389/fpubh.2016.00086 27200338 PMC4854872

[70] Witt A, Matthews V, Bailie R, Valery PC, Adams J, Garvey G, Martin JH, Cunningham FC. Aboriginal and Torres Strait Islander patients’ cancer care pathways in Queensland: Insights from health professionals. Health Promotion Journal of Australia; 2021. DOI: 10.1002/hpja.556 34767657

[71] Jaenke R, Butler TL, Condon J, Garvey G, Brotherton JM, Cunningham J, Anderson K, Tong A, Moore SP, Whop LJ. Health care provider perspectives on cervical screening for Aboriginal and Torres Strait Islander women: a qualitative study. Australian and New Zealand Journal of Public Health. 2021; 45: 150–7. DOI: 10.1111/1753-6405.13084 33683744

[72] Carr T, Arcand L, Roberts R, Sedgewick J, Ali A, Groot G. The experiences of Indigenous people with cancer in Saskatchewan: a patient-oriented qualitative study using a sharing circle. CMAJ open. 2020; 8: E852–E9. DOI: 10.9778/cmajo.20200012 PMC7881746 33303571

[73] Ciccone N, Armstrong E, Adams M, Hersh D, McAllister M, Bessarab D, Godecke E, Coffin J, Walley M. Yarning together: Incorporating telehealth into the provision of culturally secure speech pathology services for Aboriginal Australians after brain injury. Brain Impairment. 2020; 21(SUPPL 3): 328.

[74] Campbell S, Roux N, Preece C, Rafter E, Davis B, Mein J, Boyle J, Fredericks B, Chamberlain C. Paths to improving care of Australian Aboriginal and Torres Strait Islander women following gestational diabetes. Primary Health Care Research & Development. 2017; 18: 549–62. DOI: 10.1017/S1463423617000305 28714432

[75] Conway J, Lawn S, Crail S, McDonald S. Indigenous patient experiences of returning to country: a qualitative evaluation on the Country Health SA Dialysis bus. BMC Health Services Research. 2018; 18: 1010. DOI: 10.1186/s12913-018-3849-4 30594208 PMC6311048

[76] Zubrzycki J, Shipp R, Jones V. Knowing, Being, and Doing: Aboriginal and Non-Aboriginal Collaboration in Cancer Services. Qualitative Health Research. 2017; 27: 1316–29. DOI: 10.1177/1049732316686750 28682709 PMC5502907

[77] Carey TA, Schouten K, Wakerman J, Humphreys JS, Miegel F, Murphy S, Arundell M. Improving the quality of life of palliative and chronic disease patients and carers in remote Australia with the establishment of a day respite facility. BMC Palliative Care. 2016; 15: 62. DOI: 10.1186/s12904-016-0136-1 27430257 PMC4950073

[78] Taylor EV, Lyford M, Holloway M, Parsons L, Mason T, Sabesan S, Thompson SC. “The support has been brilliant”: experiences of Aboriginal and Torres Strait Islander patients attending two high performing cancer services. BMC Health Services Research. 2021; 21: 493. DOI: 10.1186/s12913-021-06535-9 34030670 PMC8142293

[79] Kirkham R, King S, Graham S, Boyle JA, Whitbread C, Skinner T, Rumbold A, Maple-Brown L. ‘No sugar’, ‘no junk food’, ‘do more exercise’ – moving beyond simple messages to improve the health of Aboriginal women with Hyperglycaemia in Pregnancy in the Northern Territory – A phenomenological study. Women and Birth. 2021; 34(6): 578–84. DOI: 10.1016/j.wombi.2020.10.003 33144033

[80] Arora RD. Salient features of an indigenous integrated inpatient model of delivery of supportive medicine services: A narrative review. Annals of Oncology. 2018; 29(Supplement 8): viii638. DOI: 10.1093/annonc/mdy300.114

[81] Eadie S, Tane M. Making a difference through partnership; heart guide aotearoa, increasing uptake and completion of cardiac rehabilitation. Heart Lung and Circulation 1. 2010; S9. DOI: 10.1016/j.hlc.2010.04.018

[82] Chamberlain-Salaun J, Mills J, Kevat P M, Remond MGW, Maguire GP. Sharing success – understanding barriers and enablers to secondary prophylaxis delivery for rheumatic fever and rheumatic heart disease. BMC Cardiovascular Disorders. 2016; 16(1): (no pagination). DOI: 10.1186/s12872-016-0344-x PMC5007824 27581750

[83] Kim J, Driver DD. Teleophthalmology for first nations clients at risk of diabetic retinopathy: a mixed methods evaluation JMIR Medical Informatics. 2015; 3: e10. DOI: 10.2196/medinform.3872 25705814 PMC4376131

[84] Wettasinghe PM, Allan W, Garvey G, Timbery A, Hoskins S, Veinovic M, Daylight G, Mack HA, Minogue C, Donovan T, Broe GA, Radford K, Delbaere K. Older Aboriginal Australians’ Health Concerns and Preferences for Healthy Ageing Programs. International Journal of Environmental Research & Public Health [Electronic Resource]. 2020; 17: 10. DOI: 10.3390/ijerph17207390 PMC7600369 33050541

[85] Govil D, Lin I, Dodd T, Cox R, Moss P, Thompson S, Maiorana A. Identifying culturally appropriate strategies for coronary heart disease secondary prevention in a regional Aboriginal Medical Service. Australian Journal of Primary Health. 2014; 20: 266–72. DOI: 10.1071/PY12117 23755824

[86] Atkinson A, Ford S, Gock H, Ierino F, Goodman D. Overcoming barriers for indigenous australians gaining access to the kidney transplant list. Nephrology. 2018; 23(Supplement 3): 49–50.

[87] Gonzalez T, Harris R, Williams R, Wadwell R, Barlow-Stewart K, Fleming J, Buckman M. Exploring the barriers preventing Indigenous Australians from accessing cancer genetic counseling. Journal of Genetic Counseling. 2020; 29: 542–52. DOI: 10.1002/jgc4.1251 32173983

[88] Hughes JT, Freeman N, Beaton B, Puruntatemeri A-M, Hausin M, Tipiloura G, Wood P, Signal S, Majoni SW, Cass A, Maple-Brown LJ, Kirkham R. My experiences with kidney care: A qualitative study of adults in the Northern Territory of Australia living with chronic kidney disease, dialysis and transplantation. PLOS ONE. 2019; 14: e0225722. DOI: 10.1371/journal.pone.0225722 31856215 PMC6922340

[89] Freene N, Brown R, Collis P, Bourke C, Silk K, Jackson A, Davey R, Northam HL. An Aboriginal and Torres Strait Islander Cardiac Rehabilitation program delivered in a non-Indigenous health service (Yeddung Gauar): a mixed methods feasibility study. BMC Cardiovascular Disorders. 2021; 21. DOI: 10.1186/s12872-021-02016-3 PMC8088627 33932992

[90] Wozniak LA, Soprovich AL, Johnson JA, Eurich DT. Adopting and implementing an innovative model to organize diabetes care within First Nations communities: A qualitative assessment. BMC Health Services Research. 2021; 21. DOI: 10.1186/s12913-021-06424-1 PMC8094479 33941176

[91] Parikh DR, Diaz A, Bernardes C, De Ieso PB, Thachil T, Kar G, Stevens M, Garvey G. The utilization of allied and community health services by cancer patients living in regional and remote geographical areas in Australia. Supportive Care in Cancer. 2021; 29: 3209–17. DOI: 10.1007/s00520-020-05839-6 33094356

[92] Eades A, Hackett ML, Liu H, Brown A, Coffin J, Cass A. Qualitative study of psychosocial factors impacting on Aboriginal women’s management of chronic disease. International Journal for Equity in Health. 2020; 19: 8. DOI: 10.1186/s12939-019-1110-3 31931810 PMC6958573

[93] Burgess C, Berwick C. Aboriginal peoples’ perceptions and beliefs about quality teaching. Australian Association for Research in Education, Freemantle; 2009.

[94] Commission S C o t P. Steering Committee for the Review of Government Service Provision Overcoming Indigenous disadvantage: key indicators 2016 report. In: Overcoming Indigenous disadvantage: key indicators, (Canberra: Steering Committee for the Review of Government Service Provision); 2016.

[95] White P, Townsend C, Cullen J, Lakhani A, White A, McIntyre M, Wright C. Understanding complex disablement among marginalized indigenous Australians: Insights from the quigley street night shelter project. Australian and New Zealand Journal of Psychiatry. 2019; 53(Supplement 1): 49.

[96] Abu-Saad K, Daoud N, Kaplan G, Ziv A, Cohen AD, Pollack D, Olmer L, Kalter-Leibovici O. A strengths-based approach to exploring diabetes management in an Indigenous minority population: A mixed methods study. PLOS ONE. 2021; 16: e0261030. DOI: 10.1371/journal.pone.0261030 34890440 PMC8664199

[97] Kitson A, Brook A, Harvey G, Jordan Z, Marshall R, O’Shea R, Wilson D. Using complexity and network concepts to inform healthcare knowledge translation. International Journal of Health Policy and Management. 2018; 7: 231. DOI: 10.15171/ijhpm.2017.79 29524952 PMC5890068

[98] Johnson RB. Dialectical pluralism: A metaparadigm whose time has come. Journal of Mixed Methods Research. 2017; 11: 156–73. DOI: 10.1177/1558689815607692

[99] Pinero de Plaza MA. PROLIFERATE: An adaptable framework with tools to evaluate different processes, outputs, and products via participatory research. figshare; 2022.

[100] Pinero de Plaza MA, Yadav L, Kitson A. Co-designing, measuring, and optimizing innovations and solutions within complex adaptive health systems. Frontiers in Health Services. 2023; 3. DOI: 10.3389/frhs.2023.1154614 PMC10103186 37063372

[101] Nct. iCARE 2.0: A Pilot Intervention of Dialectical Behavioural Therapy for Adolescents With Type 2 Diabetes; 2021. https://clinicaltrials.gov/show/NCT05107154

[102] Tipene-Leach DC, Coppell KJ, Abel S, Pahau HL, Ehau T, Mann JI. Ngati and healthy: translating diabetes prevention evidence into community action. Ethnicity & Health. 2013; 18: 402–14. DOI: 10.1080/13557858.2012.754406 23360172

[103] Gomersall JS, Gibson O, Dwyer J, O’Donnell K, Stephenson M, Carter D, Canuto K, Munn Z, Aromataris E, Brown A. What Indigenous Australian clients value about primary health care: a systematic review of qualitative evidence. Australian & New Zealand Journal of Public Health. 2017; 41: 417–23. DOI: 10.1111/1753-6405.12687 28712137

[104] Doran CM, Bryant J, Langham E, Bainbridge R, Begg S, Potts B. Scope and quality of economic evaluations of Aboriginal and Torres Strait Islander health programs: a systematic review. Australian and New Zealand Journal of Public Health. 2022; 46: 361–9. DOI: 10.1111/1753-6405.13229 35298065

[105] Zhao Y, Thomas SL, Guthridge SL, Wakerman J. Better health outcomes at lower costs: the benefits of primary care utilisation for chronic disease management in remote Indigenous communities in Australia’s Northern Territory. BMC Health Services Research. 2014; 14: 463. DOI: 10.1186/1472-6963-14-463 25281064 PMC4282496

[106] Zhao Y, Thomas SL, Guthridge SL, Wakerman J. Better health outcomes at lower costs: the benefits of primary care utilisation for chronic disease management in remote Indigenous communities in Australia’s Northern Territory. BMC health services research. 2014; 14: 1–9. DOI: 10.1186/1472-6963-14-463 25281064 PMC4282496

[107] Dalton A, Mohebbi M, Carter RA. Economic Evaluation of the Indigenous Australians ’ Health Programme Phase I 28 May 2018 Report prepared for the Department of Health; 2018.

[108] Reid P, Paine S-J, Te Ao B, Willing EJ, Wyeth E, Vaithianathan R, Loring B. Estimating the economic costs of Indigenous health inequities in New Zealand: a retrospective cohort analysis. BMJ open. 2022; 12: e065430. DOI: 10.1136/bmjopen-2022-065430 PMC9594571 36265912

[109] Barbo G, Alam S, Kiafar A. Experiences of Indigenous peoples in Canada with primary health care services: a qualitative systematic review protocol. JBI Evidence Synthesis. 2021; 19: 2398–405. DOI: 10.11124/JBIES-20-00389 34149021

[110] Lock M, Kaufert P. Menopause, local biologies, and cultures of aging. American Journal of Human Biology. 2001; 13: 494–504. DOI: 10.1002/ajhb.1081 11400220

[111] Kitson A, Feo R, Lawless M, Arciuli J, Clark R, Golley R, Lange B, Ratcliffe J, Robinson S. Towards a unifying caring life-course theory for better self-care and caring solutions: A discussion paper. Journal of Advanced Nursing. 2022; 78: e6–e20. DOI: 10.1111/jan.14887 34002886 PMC9292879

[112] Martín JG. Indigenous Health Agents in Amazonia: Creative Intermediations and a Poiesis of Care Tipití: Journal of the Society for the Anthropology of Lowland South America. 2022; 18: 1–24.

[113] Pinero De Plaza MA, Conroy T, Mudd A, Kitson A. Using a Complex Network Methodology to Track, Evaluate, and Transform Fundamental Care. Stud Health Technol Inform. 2021 Dec 15; 284: 31–35. DOI: 10.3233/SHTI210656. PMID: 34920462 34920462

[114] Conroy T, Pinero de Plaza MA, Mudd A, Mitchell M, Kitson A. Measuring fundamental care using complexity science: A descriptive case study of a methodological innovation. Journal of Clinical Nursing; 2021. DOI: 10.1111/jocn.15905 34137100

[115] Wiles LK, Kay D, Luker JA, Worley A, Austin J, Ball A, Bevan A, Cousins M, Dalton S, Hodges E, Horvat L, Kerrins E, Marker J, McKinnon M, McMillan P, Pinero De Plaza MA, Smith J, Yeung D, Hillier SL. Consumer engagement in health care policy, research and services: A systematic review and meta-analysis of methods and effects. PLOS ONE. 2022; 17: e0261808. DOI: 10.1371/journal.pone.0261808 35085276 PMC8794088

[116] Tobias JK, Tinmouth J, Senese LC, Jumah N, Llovet D, Kewayosh A, Rabeneck L, Dobrow M. Health Policy as a Barrier to First Nations Peoples’ Access to Cancer Screening. Healthcare Policy = Politiques de sante. 2020; 15: 28–46. DOI: 10.12927/hcpol.2020.26132 32176609 PMC7075447

